# Grapefruit Fiber Filled with Silver Nanowires Surface Plasmon Resonance Sensor in Aqueous Environments

**DOI:** 10.3390/s120912016

**Published:** 2012-08-31

**Authors:** Ying Lu, Cong-Jing Hao, Bao-Qun Wu, Xiao-Hui Huang, Wu-Qi Wen, Xiang-Yong Fu, Jian-Quan Yao

**Affiliations:** College of Precision Instrument and Opto-electronics Engineering, Key Laboratory of Opto-Electronics Information Technology (Ministry of Education), Tianjin University, Tianjin 300072, China; E-Mails: cathyhcj@163.com (C.-J.H.); baoqunwu@126.com (B.-Q.W.); huangxiaohui5644@gmail.com (X.-H.H.); lasertju@163.com (W.-Q.W.); fuxiangyong1234@163.com (X.-Y.F.); jqyao@tju.edu.cn (J.-Q.Y.)

**Keywords:** grapefruit photonic crystal fiber, surface plasmon resonance, silver nanowire, nanowire distance and number

## Abstract

A kind of surface plasmon resonance sensor based on grapefruit photonic crystal fiber (PCF) filled with different numbers of silver nanowires has been studied in this paper. The surface plasmon resonance modes and the sensing properties are investigated comprehensively using the finite element method (FEM). The simulation results show that the intensity sensitivity is related to nanowire numbers and the distance between two nanowires. The optimum value obtained is 2,400 nm/RIU, corresponding to a resolution of 4.51 × 10^−5^ RIU with a maximum distance of 2 μm. To a certain extent, the PCF filled with more nanowires is better than with just one. Furthermore, the air holes of grapefruit PCF are large enough to operate in practice. Moreover, the irregularity of the filled nanowires has no effect on sensitivity, which will be very convenient for the implementation of experiments.

## 1. Introduction

Surface plasmon resonance (SPR) is one of the most promising optical techniques which refers to the excitation of surface plasmon polaritons. Surface plasmons can be excited by light when the phase matching condition is met between the exciting light and the surface plasmons [1–4]. Surface plasmon resonance-based biochemical sensing has been one of the emerging new biochemical substances analysis technologies in recent years. It has more excellent features than the traditional surface analysis technology, like the fact that surface plasmon resonance-based biochemical sensing can realize real-time monitoring, with higher sensitivity, lower costs, and mini-size devices, *etc.* so SPR-based sensing has great potential for application in the field of biological material detection [5].

Photonic crystal fiber (PCF) has a lot of peculiar properties, such as endless single-mode characteristic, high birefringence coefficient, controllable dispersion, high nonlinear coefficient, *etc.* It has attracted great attention in the development of new fiber optic components [6,7]. As photonic crystal fibers have many excellent features, sensors made of them are now hot research topics in the sensing area [1].

The sensing mechanism of PCF-based SPR sensors is through coupling the leaky core mode to the plasmon to achieve resonance sensing [8]. The flexible design of the photonic crystal fiber, makes it easy to equate the effective index of the core mode to that of the material under test. Thus phase matching between the core mode and the plasmon can be easily achieved at the required wavelength and then resonance occurs. Especially, the fabrication of sensors doesn't need removing of cladding or tapering fibers as traditional fibers do. Many scientists have put forward a lot of surface plasmon resonance sensor designs based on PCFs and plenty of simulations and calculations have been made, showing the great advantages and good application prospects of these new sensors [9,10].

Metal nanoparticles and nanowires (columns), and other nanostructures can also be used to generate surface plasmon resonance [11–13]. In this paper, SPR sensors based on PCF filled with different numbers of silver nanowires have been analyzed though the finite element method (FEM) by using COMSOL Multiphysics software, regularity of the resonant wavelength changing with refractive index of the sample has been numerically simulated, and resonant wavelength detection as well as intensity detection sensitivity have also been discussed. Numerical results show that the sensitivity will increase with the increase of relative distance between two silver nanowires within 2 μm, and then it tends to be stable with the continued increase. The spectral and intensity sensitivity of the grapefruit PCF filled with more silver nanowires is better than the one filled with less. Moreover, as the air holes of grapefruit PCF are large enough, the fabrication is expected to be easy.

## 2. Numerical Simulation and Analysis

### 2.1. SPR Sensors Based on PCFs Filled with One Silver Nanowire of 300 nm Radius

**Figure 1.**
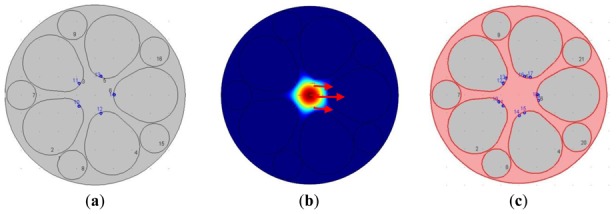
(**a**) Structure diagram of the grapefruit PCF occupied by silver nanowires (indicated by blue dots) of 300 nm; (**b**) Optical field distribution of the fundamental mode (the wavelength of the incident light is 600 nm); (**c**) Structure diagram of the grapefruit PCF occupied by two silver nanowires (è ≈ 20°).

We utilize the self-designed grapefruit PCF made of silica in the simulation [14]. The diameter of the core is approximately 10 microns and the large hole of the PCF is easy to fill for measuring fluid samples. The structure of the PCF is shown in Figure 1, with each grapefruit hole occupied by one silver nanowire of 300 nm radius. The refractive index of the fused silica optical fiber can be determined by the Sellmeier equation and for the relative dielectric constant of silver we referred to the Handbook of Optics [15]. In recent years, PCF-based SPR sensors used in refractive index sensing of aqueous analytes have attracted much attention [16,17]. All holes are filled with liquid samples (n_a_ = 1.33) in this paper.

We use the finite element method (FEM) to solve the light field mode and calculate the attenuation constant of the fundamental mode of fiber with different mosaic structures of silver nanowires (optical field distribution of the fundamental mode is shown in Figure 1(b), and the arrow indicates the polarization direction of magnetic field). The attenuation constant of the fundamental mode is calculated for different wavelengths of incident light. The wavelength with maximal transmission loss can be identified and light intensity detection sensitivity at different wavelengths can also be investigated. It is easy to prove that the power attenuation coefficient á is:

(1) α=2k0Im(neff)

where k_0_ is the wave number (k_0_ = 2ð/ë), *n_eff_* is the mode effective refractive index, the real part expressed the dispersion and the imaginary part expressed the transmission loss.

Therefore the optical fiber transmission loss coefficient can be expressed with the power attenuation coefficient á as:

(2) αloss=101ge.α=8.686⋅2πλ⋅Im[neff](dB/m)

**Figure 2.**
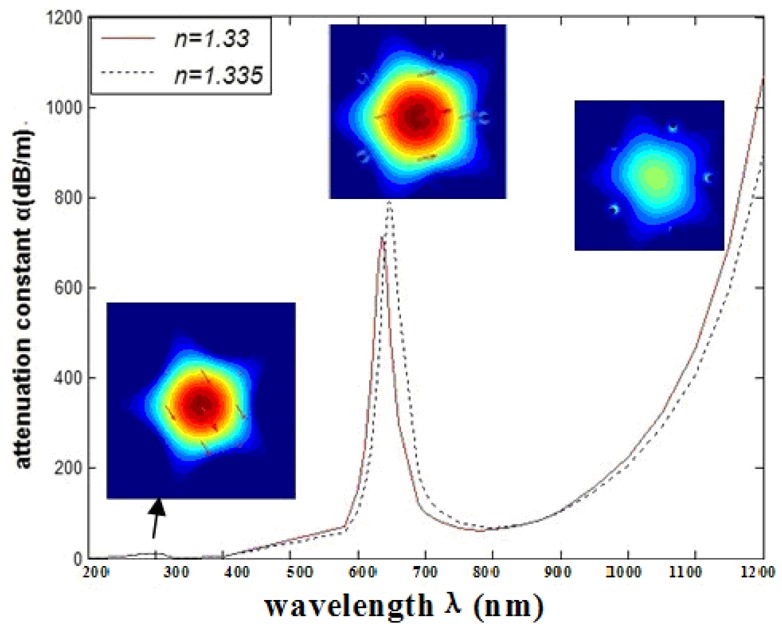
Relationship between wavelength and attenuation constant of the fundamental mode of the grapefruit PCF occupied by one silver nanowire of 300 nm radius, and the fundamental mode at peak I (the origin of the resonance peak is 310 nm) and peak II (the origin of the resonance peak is 635 nm); the red and blue curves represent the refractive indices of the samples which are 1.33 and 1.335, respectively.

This article uses the mode power attenuation coefficient *á* to quantify the loss of the transmission mode, and use dB/m as the unit. Generally, the refractive index of the analyte *n_a_* decides the resonance wavelength. When the incident light reaches the interface between two different media, it can cause metal free electronic resonance. SPR will change with the index of refraction of the surface change, so the SPR is so sensitive to environmental changes. The relationship between wavelength and attenuation constant of the fundamental mode of the grapefruit PCF filled with one silver nanowire of 300 nm radius is shown in Figure 2, we can get two loss peaks, which are located at 310 nm (peak I) and 635 nm (peak II) when n_a_ = 1.33 (red solid curve). It is noteworthy that the resonance peak shift of peak I is 1 nm, and that of peak II is 12 nm. It is obvious that the peak II is more sensitive than peak I. This can be explained by noting that the silver nanowire surface supports several waveguide modes which result in several peaks. Peak I is the coupling between the high-order mode and the core guided mode and peak II is the coupling between the fundamental waveguide mode and the core guided mode. In the next section we only focus on the peak II for sensing.

**Figure 3.**
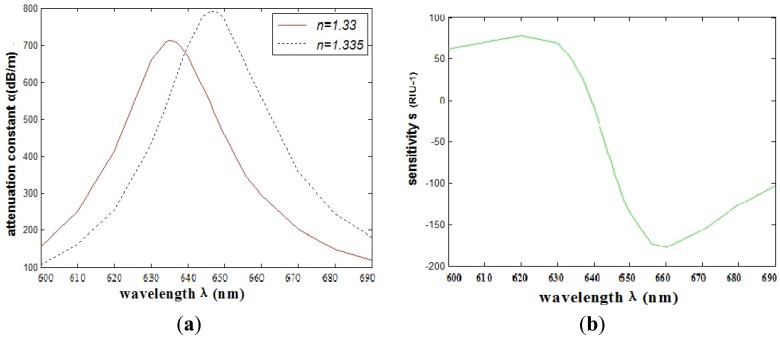
(**a**) Relationship between wavelength and attenuation constant of the fundamental mode of the grapefruit PCF occupied by one silver nanowire of 300 nm radius. The red and blue curves represent the refractive indices of the samples which are 1.33 and 1.335, respectively; (**b**) Intensity detection sensitivity (*RIU^−1^*) curve.

The relationship between wavelength and attenuation constant of the fundamental mode of the peak II is shown in Figure 3(a). The red and blue curves represent samples with refractive indexes of 1.33 and 1.335, respectively. The sharp loss peak is in the range of 630–650 nm. Because the fiber core and surface plasmon mode can produce resonance, the energy of light field in the core has a great loss. When the sample refractive index changes from 1.33 to 1.335, the resonance peak shifts 12 nm (Δ*ë*_peak_) towards the longer wavelength.

If the spectral variation of 0.1 nm can be accurately detected, we can get the corresponding refractive index resolution as [1]:

(3) $$\text{R}=\Delta n_{a}\Delta \lambda_{\min}/\Delta \lambda_{\text{peak}}=4.17\times 10^{-5}\text{RIU}$$

The power detection sensitivity for the refractive index variation *Δn_a_* can be defined as [1]:

(4) $$\text{S}(\lambda )=\frac{1}{\alpha (\lambda ,n_{a})}\frac{\partial \alpha (\lambda ,n_{a})}{\partial n_{a}}$$

We can get the maximal sensitivity at 647 nm, and equals 178 RIU^−1^. It is typically a safe assumption that a 1% change in the transmitted intensity can be detected reliably, which leads to a sensor resolution of 5.62 × 10^−5^ RIU.

### 2.2. SPR Sensors Based on PCFs Filled with Two Silver Nanowires of Different Relative Distances

**Figure 4.**
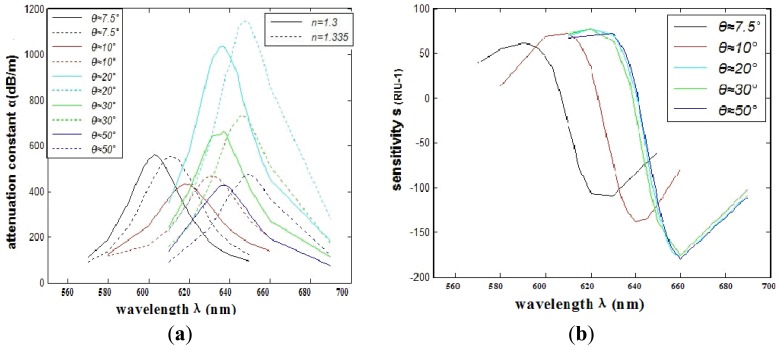
(**a**) The comparison of attenuation constant of the fundamental mode of different relative distances between two silver nanowires; (**b**) The comparison of intensity detection sensitivity *(RIU^−1^)* curves.

In consideration of the interaction between the two silver nanowires, we investigate the SPR sensors based on PCFs filled with two silver nanowires of different relative distances. Numerical simulation shows that when the number of the nanowires increases to two, the transmission loss coefficient and the sensitivity will change for the interaction of electromagnetic field between the two silver nanowires. Then the relative distance between two silver nanowires has changed. In order to make it feasible for real operation, we place the silver nanowires in the edge of the circle (as shown in Figure 1(a)) to have a certain degree rotation *è* (as shown in Figure 1(c)). The comparison of the attenuation spectra of the fundamental mode and the amplitude detection sensitivity curves is shown in Figure 4(a,b), respectively. The maximum sensitivity of the sensor reaches 2,400 nm/RIU (the sensitivity is higher than the previous value, such as the sensitivity 2,200 nm/RIU achieved by the authors of reference [1]) when the sensitivity of the sensor is defined as:

(5) $$S=\frac{\partial \lambda }{\partial n_{a}}$$

**Figure 5.**
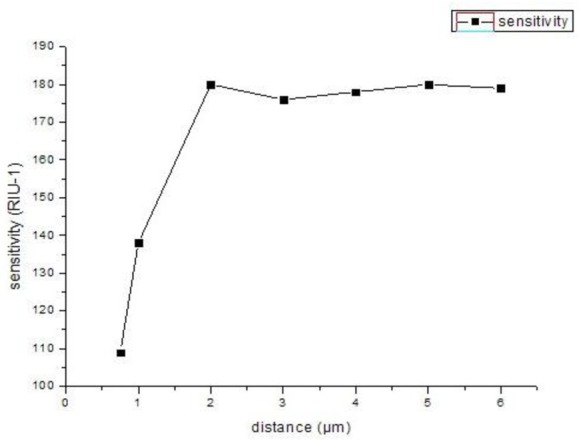
The changes of sensitivity of different relative distances between two silver nanowires in the PCF.

As shown in Figure 4, we can see that the SPR peak is sensitive to changes in the relative distances between the two silver nanowires. However, the sensitivity improvement has a limited value. As shown in Figure 5 numerical simulation results show that the sensitivity will increase (from 109 RIU^−1^ to 180 RIU^−1^) with the increase of relative distance between two silver nanowires within 2 ìm (*è* ≈ *20*°), and then it tends to be stable with the continued increase. By adjusting the distance between the two silver nanowires, we can tune the resonance wavelength to a desired value. So when performing experiments, we should control the distance betwwen the two silver nanowires.

### 2.3. SPR Sensors Based on PCFs Filled with Three Silver Nanowires

**Figure 6.**
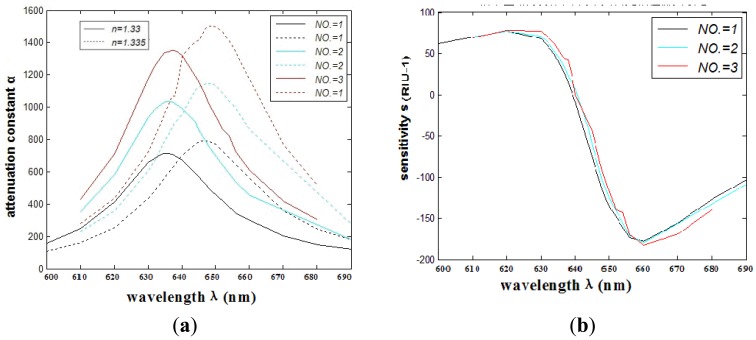
(**a**) The comparison of attenuation constant of the fundamental mode of different numbers (*NO.*) of silver nanowires; (**b**) The comparison of intensity detection sensitivity *(RIU^−1^)* curves.

When the number of the nanowires increases to three, the transmission loss coefficient and the sensitivity will change for the interaction of electromagnetic field between the silver nanowires. The results show that the spectral and intensity sensitivity (183 RIU^−1^) of the grapefruit PCF filled with more silver nanowires (within three) is better than the one filled with less. The sensor resolution reaches 5.46 × 10^−5^ RIU. The comparisons of attenuation spectra of the fundamental mode and the amplitude detection sensitivity curves are shown in Figure 6(a,b), respectively.

**Figure 7.**
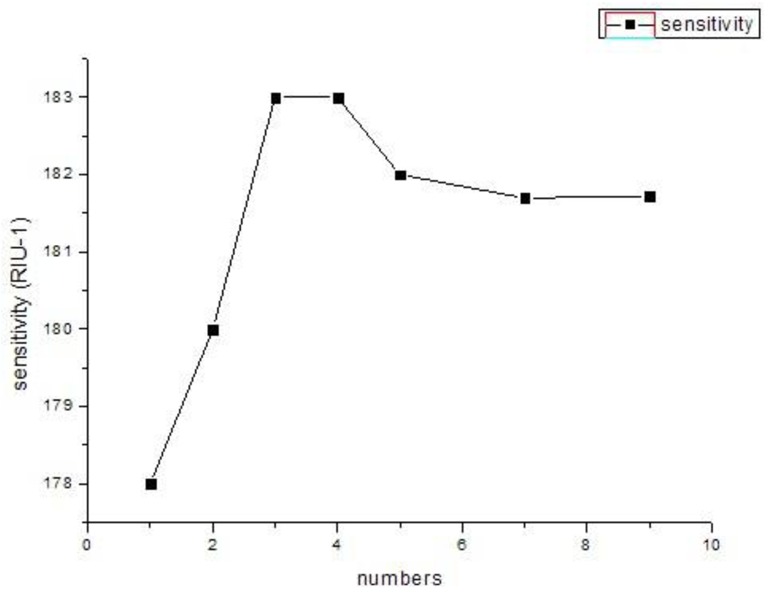
The relationship between the sensitivity and different numbers of PCFs filled with silver nanowires.

Meanwhile, as shown in Figure 7, the sensitivity will remain relatively stable with the continuous increase in the number of silver nanowires. We can explain this by noting that the increase of the numbers of silver nanowires has no enhanced coupling between the plasmonic mode and core-guided mode. So with this numerical result, better results are expected to be obtained filling with nanowires, which is more convenient to operate in practice than using nanowires. It can overcome the difficulties of coating the holes in the photonic crystal fiber and the control of the distance between the silver nanowires.

### 2.4. Grapefruit PCFs Filled with Three Silver Nanowires Irregularly

**Figure 8.**
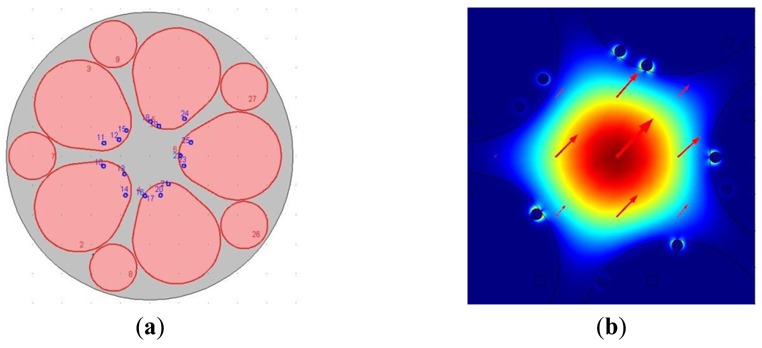
(**a**) The surface plasmon resonance sensors based on grapefruit PCF irregularly filled with three silver nanowires; (**b**) Calculated magnetic field distribution and Im (*n_eff_*) = 5.148529e-5i of the guided-mode that can excite and resonant with plasmonic modes (the wavelength of the incident light is 649 nm, n_a_ = 1.335).

Based on practical considerations, such as that it can be more close to the condition of that the holes filled with three silver nanowires irregularly during the actual operation, we carried out the related simulation experiment of the surface plasmon resonance sensors based on grapefruit PCF irregularly filled with three silver nanowires, and the resonant mode obtained is shown in Figure 8.

**Figure 9.**
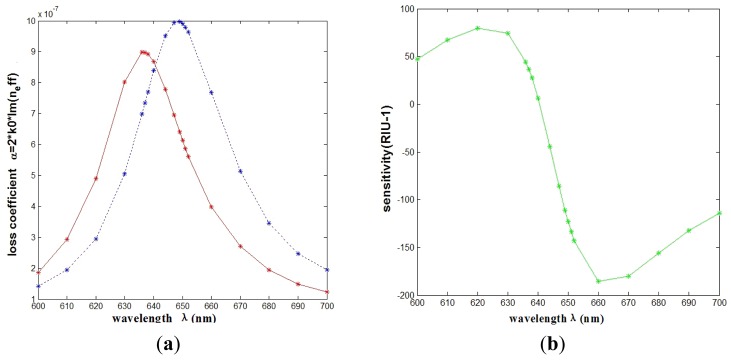
(**a**) The curves of attenuation constant of the fundamental mode of the grapefruit PCF irregularly filled with three nanowires. The red and blue curves represent the refractive indices of the samples are 1.33 and 1.335, respectively; (**b**) Intensity detection sensitivity *(RIU^−1^)* curve.

The numerical results show that it is the same Δ*ë*_peak_ (=12 nm) as the regular ones. The relationship between wavelength and attenuation constant of the fundamental mode of the grapefruit PCF filled with three silver nanowires irregularly is shown in Figure 9(a) (the wavelength range is 600–700 nm, *n_a_* = 1.33, 1.335). Regarding the intensity detection sensitivity, it 185 RIU^−1^, which is also similar to the surface plasmon resonance sensors based on grapefruit PCF regularly filled with three silver nanowires (183 RIU^−1^), as shown in Figure 9(b), so we can note that it almost has no effect for the grapefruit PCF irregularly filled with three silver nanowires. It is similar to that the sensitivity tends to be stable with the continued increase of the distance (>2 ìm) between the nanowires, so for the actual operation [the grapefruit PCF irregularly filled with three silver nanowires (>2 ìm)], we need not to pursue the regularity deliberately. This will be very convenient for the implementation of the experiments.

## 3. Conclusions

In this paper we have analyzed surface plasmon resonance sensors based on photonic crystal fibers filled with silver nanowires of different numbers and different distances between two silver nanowires through the finite element method (FEM) by using the COMSOL Multiphysics software. Numerical results show that the sensitivity will increase from 109 RIU^−1^ to 180 RIU^−1^ with the increase of relative distance between two silver nanowires within 2 ìm (è ≈ 20°), and then it tends to be stable with the continued increase. With the appropriate distance between two silver nanowires, when the number increases to three, the spectral and intensity sensitivity is better (from 178 RIU^−1^ to 183 RIU^−1^) due to the interaction of electromagnetic field between the silver nanowires. The sensitivity will remain relatively stable with the continuous increasing of the numbers of silver nanowires and the PCF filled with more nanowires is better than the one. Thus by adjusting the distance between the two silver nanowires and the numbers of silver nanowires, we can tune the resonance wavelength to a desired value. Moreover, the irregularity of the filled nanowires has no effect on sensitivity, which will be very convenient for the practical implementation of the experiment.
